# Mean platelet volume and total white blood cells as diagnostic biomarkers for acute appendicitis at Omdurman military hospital: a cross-sectional study − 2021

**DOI:** 10.1186/s12876-023-03091-w

**Published:** 2023-12-16

**Authors:** Mohamed Osman Suliman Basher, Ahmed Abdelfattah Ahmed, Aamir Abdullahi Hamza

**Affiliations:** 1 Department of General Surgery, Omdurman Military Hospital, Alneelain University, Khartoum, Sudan; 2 Department of General Surgery, Omdurman Teaching Hospital, College of Medicine, University of Bahri, Khartoum, Sudan

**Keywords:** Appendicitis, Diagnosis, MPV, Diagnostic biomarkers

## Abstract

**Background:**

Acute appendicitis (AA) is among the most common nontraumatic indications for emergent abdominal surgical procedures. However, accurately diagnosing all cases can be challenging, physical examination, biochemical markers, and imaging techniques can sometimes be insufficient. This study aimed to (a) compare the mean platelet volume (MPV) and total white blood cell count (TWBCs) in patients with or without histological evidence of an inflamed appendix and (b) determine the positive predictive value, negative predictive value, sensitivity, and specificity of both MPV and TWBCs as diagnostic biomarkers in the diagnosis of AA. We conducted this research at Omdurman Military Hospital, focusing on patients who presented with symptoms suggestive of AA.

**Materials and methods:**

An analytical cross-sectional study conducted at Omdurman Military Hospital from December 2020 - December 2021. The study population was patients who presented to our emergency department with symptoms and signs suggestive of AA. Participants were patients operated on as cases of AA. Data variables included sociodemographic characteristics, clinical presentations, intraoperative appendix macroscopic findings, preoperative MPV and total white blood cell count (TWBCs), and postoperative histopathological findings.

**Results:**

A total of 106 patients were included in this study, with 75 (68.2%) males; half were 10–19 years old. Sixty-three patients (57.3%) had low (< 7.5 fL) MPV, whereas 47 (42.7%) had normal values. Comparing MPV to total white blood cells (TWBCs) revealed that MPV was more sensitive (84.6%) and specific (90%) than TWBCs during the first 24 h of inflammation. However, the TWBCs were more sensitive (97.2%) but less specific (94.7% vs. 100%) after 24 h of onset.

**Conclusion:**

In this study, MPV was lower in patients with AA, while there was an increase in TWBCs. The high sensitivity and specificity of TWBCs and MPV indicated that they form a promising diagnostic marker for AA.

**Supplementary Information:**

The online version contains supplementary material available at 10.1186/s12876-023-03091-w.

## Introduction

Acute appendicitis (AA) is arguably the most prevalent surgical emergency encountered globally [1]. In 2019, 228 AA cases were diagnosed per 100,000 people, and there were an estimated 17.7 million cases worldwide. Over 33,400 deaths occurred in the same year, making up 0.43 fatalities for every 100,000 people [2].

In surgical practice, diagnosing AA is considered difficult since it requires integrating clinical, laboratory, and radiographic evidence. Clinical scoring methods incorporating physical exam findings and inflammatory markers are continually used to improve diagnostic accuracy [3].

MPV is a measure of platelet size generated by full blood count analysers as part of the routine complete blood count (CBC) test cycle, which is commonly overlooked by clinicians [4]. MPV can provide crucial information on the course and prognosis of many pathological conditions, such as cardiovascular diseases, respiratory diseases, Crohn’s disease, rheumatoid arthritis, juvenile systemic lupus erythematosus and diabetes mellitus [5–8]. Many researchers have discussed the introduction of MPV as a diagnostic biological marker for the diagnosis of AA to help increase the accuracy of the diagnosis [4].

Many studies have investigated the correlation between AA and MVP; however, their conclusions could be more consistent, with variations in the low and elevated levels [9]. This study aimed to (a) compare the MPV and total white blood cells (TWBCs) in patients with or without histological evidence of an inflamed appendix and (b) determine the positive predictive value, negative predictive value, sensitivity, and specificity of MPV as a diagnostic biomarker in the diagnosis of AA.

## Materials and methods

### Study settings

This study was conducted at the emergency department of Omdurman Military Hospital in Omdurman, Khartoum, Sudan, from December 2020 to December 2021 using a cross-sectional design.

We conducted a comprehensive data collection, ensuring total coverage of the target population within one hospital center over a one-year period.

### Patients

The study population consisted of patients who presented to our emergency department with symptoms and signs suggestive of AA. Participants were patients operated on as cases of AA. Patients with ectopic pregnancy, ovarian cyst, age < ten years, those diagnosed with heart failure, peripheral vascular disease, hematological disorders, acute or chronic infection, cancer, ulcerative colitis, rheumatoid, hepatic diseases and hematological diseases, patients who received anticoagulants, nonsteroidal anti-inflammatory drugs or oral contraceptives, and patients who received a blood transfusion in the last group were excluded.

Data variables included sociodemographic characteristics, clinical presentations, intraoperative appendix macroscopic findings, preoperative MPV and TWBCs, and postoperative histopathological findings.

MPV and TWBCs for all patients were analysed at our hospital laboratory, and the normal values were determined according to our laboratory reference to be (4.5 × 10^3/mm^3–11 × 10^3/mm^3) for TWBCs and 7.5 fL – 12 fL for MPV. Data were analysed using the Statistical Package for Social Sciences (SPSS) computer program version 24.0. Descriptive statistics were used; mean ± standard deviation (SD) for continuous variables and frequency, percent for categorical variables. Patients’ appendices were categorized according to their postoperative histopathological findings into inflamed and noninflamed, which were then cross-tabulated with normal (7.5 fL to 12 fL) and low (< 7.5 fL) categories of MVP and normal (4.5 × 10^3/mm^3–11 × 10^3/mm^3) and high (> 11 × 10^3/mm^3) categories of TWBCs. Patients were also categorized according to the onset of symptoms into those who presented within 24 h of symptom onset and those who presented in more than 24 h. The chi-square test was used to examine differences between groups, and a *P* value of < 0.05 was considered to indicate statistical significance.

## Results

### Sociodemographic characteristics

This study included 106 patients, primarily males, with 75 (70.7%). The average age was [15.5 ± 25.93], with half (50%) of the patients in the age group 10–19 years.

### Signs and symptoms

The common triad of signs was fever, rebound tenderness, and tenderness, observed in 98.2%, 88.8%, and 83.6% of patients, respectively. Guarding and rigidity were less frequent, noted in 25.5% and 23.6% of patients, respectively. Nearly half of the patients, 54 (50.9%), presented within 24 h of onset.

### Medications and imaging

Preoperatively, 17 (16%) patients received antibiotics, 28 (26.4%) received analgesics, and nine (8%) patients were given antispasmodics. Abdominal ultrasound was performed on 103 (93.6%) patients, while 18 (16.3%) underwent abdominal CT scans. All patients who underwent CT also had an abdominal ultrasound.

### Outcome

Intraoperatively, the appendix was found to be inflamed in 63 (59.4%) patients, not inflamed in 21 (19.8%), showed an appendicular abscess in 7 (6.6%), and was perforated in 15 (14.1%) patients. Histopathology of the specimens revealed that acute inflammation was present in the majority, with 87 patients accounting for 82%.

### Findings of the inflammatory markers

The mean TWBCs among the patients was 12.3 ± 3.6, with 54.5% having a high TWBCs count (> 11 × 10^3/mm^3). However, the MPV had a mean value of 7.6 ± 0.8 fL, with 63 (57.3%) patients showing low (< 7.5 fL) readings (Table 1).

**Table 1:** TWBCs and MPV of patients who presented with AA at Omdurman Military Hospital

Variables	Frequency	Percent
TWBCs		
Normal (4.5–11 × 10^3/mm^3)	46	43.4
High (> 11 × 10^3/mm^3)	60	56.6
MPV		
Normal (7.5- 12fL)	43	42.7
Low (< 7.5fL)	63	57.3

Mean ± SD: TWBCs: 12.3 ± 3.6, MPV: 7.6 ± 0.8

In a comparison between patients showing histological signs of an inflamed appendix and those without, a significant proportion of the former group exhibited elevated TWBCs (68%, 59 out of 87) and reduced MPV (71.2%, 62 out of 87), contrasting markedly with those having a noninflamed appendix. This difference was statistically significant (*p* value < 0.05) (Table 2).

**Table 2:** Comparison of MPV and TWBCs between patients with postoperative histological evidence of inflamed appendix and those without

Variables	Inflamed (n = 87)	Noninflamed (n = 19)	Total (n = 106)	Chi-Square *χ*2	*p* value
TWBCs					
Normal	28 (32.2%)	18 (94.7%)	46	24.8	< 0.001*
High	59 (67.8%)	1 (5.3%)	60		
MPV					
Low	62 (71.2%)	1 (5.3%)	63	28.2	< 0.001*
Normal	25 (28.8%)	18 (94.7%)	43		

* *p* value < 0.001 is considered statistically significant

Both groups of patients, those presented in < 24 h and those presented afterwards, showed the same results. Patients with an inflamed appendix demonstrated higher TWBCs and lower MPV than those with a noninflamed appendix (*P* value < 0.05) (Table 3).

**Table 3:** MPV and TWBCs in relation to histopathologically inflamed appendix and onset of symptoms

Onset of Symptoms	Parameter	Inflamed	Noninflamed	Total	Chi-Square *χ*2	*p* value
< 24 h (n = 50)	TWBCs - Normal	18 (36.0%)	11 (22.0%)	29	10.2	< 0.001
	TWBCs - High	21 (42.0%)	00 (00.0%)	21		
	MPV - Low	33 (66.0%)	01 (02.0%)	34	22.4	< 0.001
	MPV - Normal	06 (12.0%)	10 (20.0%)	16		
> 24 h (n = 56)	TWBCs - Normal	10 (18.0%)	07 (12.5%)	17	14.4	< 0.001
	TWBCs - High	38 (68.0%)	01 (01.7%)	39		
	MPV - Low	29 (52.0%)	00 (00.0%)	29	10.0	< 0.002
	MPV - Normal	19 (34.0%)	08 (14.2%)	27		
Total	TWBCs				24.8	< 0.001
	MPV				28.1	< 0.001

### Sensitivity and specificity analysis

MPV was more sensitive (84.6%) and specific (90%) than TWBCs during the first 24 h of inflammation, with 97.1% positive predictive value, 62.5% negative predictive value, and 86% accuracy. In contrast, TWBCs were more sensitive (97.2%) but not more specific (94.7% compared to 100%) after 24 h of inflammation onset, with 80.3% accuracy. (Table 4).

**Table 4:** The average sensitivity analysis of inflammatory markers in patients who presented with AA at Omdurman Military Hospital

Test	< 24 h (n = value)	> 24 h (n = value)	Total (n = value)
	TWBCs	MPV	TWBCs
Sensitivity	53.8%	84.6%	97.2%
Specificity	00.0%	90.0%	87.5%
Positive predictive value	100%	97.1%	97.4%
Negative predictive value	00.0%	62.5%	41.2%
Accuracy	64.0%	86%	80.3%

## Discussion

AA is the most common indication for emergency abdominal surgery worldwide, with a lifetime incidence of 7% [10]. It can occur at any age, with a peak incidence in the mid-twenties. Although AA is one of the most common surgical emergencies, its exact causes are still unknown [11]^,^ and no accurate diagnostic tool has been established yet. The previous traditional methods for diagnosing AA, such as clinical history, physical examination, and laboratory tests, needed to provide sufficient and accurate data to confirm the diagnosis of AA in the early stages. Radiological investigations such as computed tomography and ultrasonography are promising for early diagnosis, but they still need to be sufficiently accurate in diagnosing AA [12].

In our study, we included 106 patients who presented with AA features, with the majority presenting with right iliac fossa pain (98.2%, n = 108), 88 (80%) having rebound tenderness, and 94 (85.5%) having nausea, anorexia, and vomiting. Although 106 patients underwent an emergency appendectomy, only 87 (82%) were found to have histopathological evidence of an inflamed appendix, which emphasizes the previously mentioned need for a more accurate tool for diagnosing AA.

### MPV in acute appendicitis

Several studies have investigated the diagnostic value of laboratory inflammatory markers such as MPV in diagnosing AA and observed a decrease in MPV in this case [11, 13, 14]. Similar to these studies, we found a significant result (*p* value = < 0.001) in which 87 (82%) of the patients had low (< 7.5 fL) MPV in inflamed appendicitis compared to noninflamed ones, especially in the first 24 h of the presentation. The significance in MPV between those inflamed and healthy ones continued even after 24 h (*p* value = 0.002).

Contrary to these findings, Uyanik [15] observed no significant decrease in MPV in patients with AA. In contrast, Narci et al. [16] and Aktimuret et al. reported significantly higher MPV in AA patients [17]. The sensitivity and specificity of MPV in the diagnosis of AA were 84.6% and 90% in the first 24 h, respectively, and the sensitivity decreased in the next 24 h (60%), where the specificity rose to 100%. One study [18] showed that 63% of patients seek medical help for AA two days after the onset of symptoms. Others [19] found that the mean duration of symptoms before seeking medical attention was 3.7 days and 4 ± 3.5 days, respectively. These findings indicate that normal MPV counts could accurately rule out the presence of AA.

### TWBCs in acute appendicitis

Previous studies also showed interest in comparing the TWBCs count between AA patients and healthy individuals. Egemen Kucuk et al. [14] found that the leukocyte count was 14.3 ± 2.99 × 10^3^/mm^3^, which is significantly higher than that of healthy individuals, and it has a sensitivity and specificity of 94% and 75%, respectively. In the present study, TWBCs count also appeared to be significantly higher (*p* value = < 0.001) in patients with AA, with a mean of 12.3 ± 3.6 × 10^3^/mm3. Unlike the previous study, leukocytes had lower sensitivity and higher specificity (67.8% and 94.7%) in diagnosing AA. The same low sensitivity and high specificity of leukocytes have been reported in a previous study, where the sensitivity and specificity were found to be 67-97.8%, respectively [17]. TWBCs have been reported to be the first laboratory measure to indicate inflammation of the appendix, and several previous studies [18, 19] indicated that most patients with AA present with leukocytosis.

### Limitations of the study

Although this study adds informative results regarding MPV count in AA, it also carries some limitations. The relatively small sample of patients included in the study was considered a study limitation. The accuracy of the diagnostic methods, such as computed tomography, ultrasonography, histopathology, and physical examination, could form a limitation for this study. The current study did not examine all age groups.

## Conclusion

In this study, MPV was lower in patients with AA, while there was an increase in TWBCs counts. TWBCs and MPV both had high sensitivity and specificity, indicating that when used in combination with TWBCs count, MPV can provide valuable insight for clinicians and aid in more accurate and early diagnosis of AA, especially in low-resource settings.

However, relying solely on these markers is not recommended, as clinical judgment, patient history, and other diagnostic tests should also be considered for a comprehensive diagnosis. Further studies are needed to confirm our findings and to better understand the role of MPV in diagnosing AA.

### Electronic supplementary material

Below is the link to the electronic supplementary material.

Supplementary Material 1

## Data Availability

The dataset analysed in this study is accessible and will be provided as supplementary material.

## Abbreviations

AA: Acute appendicitis
MPV: Mean platelet volume
TWBCs: Total white blood cells
